# A New Method of Treating the Insane

**Published:** 1893-10-14

**Authors:** 


### "A New Method of Treating the Insane."

"Hop Dance cries in Tom's belly for two white herring. Croak not, black angel: I have no food for thee." Shakespeare's madman was much to be pitied, but his condition of mind (and body) was peace, perfect peace compared with that of the Irishman recorded by Mr. Stead. This unfortunate man believed himself to be the abode of the spirits of two of his fellow-countrymen, a Nationalist and an Orangeman. They occupied different sides of his body, made him their Donnybrook, and struggled for the mastery. Who would not rather lodge Hop Dance with his craving for fish than two wild Irishmen? The story of this crazy Paddy has been sufficient in these sceptical days to convince a medical man, Dr. C. Williams, Physician to the Psychic Hospital, Liverpool," how very rational is his own (and the suffering Irishman's) theory of insanity, and, moreover, he is certain that a perusal of the tale would have a similar effect upon any impartial reader. This avowal, in Dr. Williams' pamphlet under the above title, fills us with wonder at the extent of his credulity, if nothing else. He has sat at the feet of Messrs. Haweis and Stead, and is now certain that many so-called lunatics "are not mad but only obsessed"; that they are possessed by evil spirits. He has come to the conclusion that in our search after the cause, and our blind gropings after the treatment of insanity, we have lost our way in these new dark ages of the nineteenth century. The Roman and Greek priests knew more about the disease than could be gleaned from the collective wisdom of the whole Medico-Psychological Association. Yet it is no wonder that pathologists have been unable to throw a perfect light on the subject, nor can they be blamed because even with all the resources of modern science at their disposal they have not discovered this cause of madness. It was not there, and therefore they did not find it. The evil spirit had disappeared and had left no trace behind.

The poor people with suicidal or homicidal impulses are not necessarily insane (Dr. W. tells us), but "are possessed by some evil spirit or demon, who inspires the poor victim with these malevolent impulses and insane ideas." Such individuals are not fit cases for an asylum, but should rather be sent to the Psychic Hospital. The unfortunate medical man when certifying lunatics has ever the dread of the Law Courts before his eyes, but a new terror now hangs over his head. He is gravely told that hallucinations of sight and hearing are no test of insanity, and he is solemnly warned to take heed lest he consign a clairvoyant or clairaudient to a terrible fate—"for incarceration with the genuinely insane will of course tend to upset the mental balance of one previously sane," though able to see visions and converse with "the invisibles." Can it be possible that association with those suffering from mental disorders in a psychic hospital might have a similar disastrous effect?

Dr. Williams pleads for help to carry out his new and rational treatment. He wishes to have a suitable building in which to set up in business as a caster-out of devils. This method of treatment was, he tells us, practised long ago by Egyptian priests, but he does not give us any details as to how it is to be carried out. Probably he would exclaim—

> Was not all the knowledge
> Of the Egyptians writ in mystic symbols?

But why does he not unfold the mysteries? Can it be—

> Because the simple idiot should not learn it
> And make it vulgar?

An efficacious mist. diabol. would be a valuable addition to our pharmacopæia. Perhaps, however—

> He deals all
> With spirits he: he will not hear a word
> Of Galen or his tedious recipes.

We remember how the man who, with his wine, had swallowed the stingy burgomaster's soul, managed to relieve himself of his tormentor. Will Dr. Williams use such means? Can tartarated antimony or mist. sennæ co. hold the field against mystic rites or noisy tom-toms?

We look for great results from this revival. With Dr. Williams as demon-finder, Mr. Haweis as exorcist, and Mr. Stead to chronicle their doings, what might we not expect? No pathological researches (let the Lunacy Commission mourn in silence) can be looked for; but every page of the case books will be adorned with photographs of baffled fiends (taken by Mr. Haweis) just driven from their resting places. These deserted abodes will be forthwith swept and garnished. The rejected demons must be caught and bottled. This must be insisted upon by the sanitary authority in whose district the new hospital is placed. If a paltry comma-bacillus taxes to the utmost the watchfulness and skill of our health officers, what could the latter do to guard the community from a vindictive horde of evicted demons whose claims to compensation have been entirely overlooked.

